# Intraoperative adverse events among surgeons in Singapore: a multicentre cross-sectional study on impact and support

**DOI:** 10.1186/s12913-024-10998-x

**Published:** 2024-04-24

**Authors:** Clyve Yu Leon Yaow, Qin Xiang Ng, Ryan Ian Houe Chong, Clarence Ong, Nicolette Zy-Yin Chong, Nicole Li Xian Yap, Ashley Shuen Ying Hong, Benita Kiat Tee Tan, Amos Hong Pheng Loh, Andrew Siang Yih Wong, Hiang Khoon Tan

**Affiliations:** 1 NUS Yong Loo Lin School of Medicine, National University of Singapore, Singapore, Singapore; 2 Health Services Research Unit, Singapore General Hospital, Singapore, Singapore; 3 Saw Swee Hock School of Public Health, National University of Singapore and National University Health System, Singapore, Singapore; 4 Department of General Surgery, Sengkang General Hospital, Singapore, Singapore; 5 Department of Paediatric Surgery, KK Women’s and Children’s Hospital, Singapore, Singapore; 6 Department of Surgery, Changi General Hospital, Singapore, Singapore; 7 Division of Surgery and Surgical Oncology, Singapore General Hospital and National Cancer Centre Singapore, Singapore, Singapore; 8 Singhealth Duke-NUS Global Health Institute, Singapore, Singapore; 9 Duke Global Health Institute, Duke University, Durham, North Carolina, United States

**Keywords:** Adverse events, Emotional response, Intraoperative complications, Mental health

## Abstract

**Background:**

It is known that many surgeons encounter intraoperative adverse events which can result in Second Victim Syndrome (SVS), with significant detriment to their emotional and physical health. There is, however, a paucity of Asian studies in this space. The present study thus aimed to explore the degree to which the experience of an adverse event is common among surgeons in Singapore, as well as its impact, and factors affecting their responses and perceived support systems.

**Methods:**

A self-administered survey was sent to surgeons at four large tertiary hospitals. The 42-item questionnaire used a systematic closed and open approach, to assess: Personal experience with intraoperative adverse events, emotional, psychological and physical impact of these events and perceived support systems.

**Results:**

The response rate was 57.5% (*n* = 196). Most respondents were male (54.8%), between 35 and 44 years old, and holding the senior consultant position. In the past 12 months alone, 68.9% recalled an adverse event. The emotional impact was significant, including sadness (63.1%), guilt (53.1%) and anxiety (45.4%). Speaking to colleagues was the most helpful support source (66.7%) and almost all surgeons did not receive counselling (93.3%), with the majority deeming it unnecessary (72.2%). Notably, 68.1% of the surgeons had positive takeaways, gaining new insight and improving vigilance towards errors. Both gender and surgeon experience did not affect the likelihood of errors and emotional impact, but more experienced surgeons were less likely to have positive takeaways (*p* = 0.035). Individuals may become advocates for patient safety, while simultaneously championing the cause of psychological support for others.

**Conclusions:**

Intraoperative adverse events are prevalent and its emotional impact is significant, regardless of the surgeon’s experience or gender. While colleagues and peer discussions are a pillar of support, healthcare institutions should do more to address the impact and ensuing consequences.

**Supplementary Information:**

The online version contains supplementary material available at 10.1186/s12913-024-10998-x.

## Introduction

Throughout their careers, many surgeons encounter intraoperative adverse events. In the most severe instances, these incidents can lead to Second Victim Syndrome (SVS), which is a serious consequence arising from adverse patient events or medical errors that traumatise healthcare providers [1, 2]. It is estimated that around 50% of healthcare providers have experienced SVS at some point of their careers [3, 4], underscoring its significance as a pervasive issue in the healthcare field. While the causes of SVS vary, its effects are notably more pronounced amongst specific groups, including surgeons, anaesthesiologists, paediatricians, obstetricians, and gynecologists [5].

SVS exhibits varying presentations amongst individuals. Its impacts encompass psychological, physical, and professional aspects, constituting a substantial burden on medical practitioners [6, 7]. Examples include clinical depression, feelings of negativity, self-doubt, physical ailments, and work-related repercussions [6, 7]. For some, this may also be a transformative encounter, with enduring influence on both their personal lives and clinical vocations [4, 8]. Surgeons still remain a relatively under-research cohort despite the fact that they frequently confront high-stress scenarios and professional hurdles, and are presumably at heightened susceptibility to SVS. Existing studies predominantly focus on physicians [3, 4], inadvertently perpetuating the prevalent, but unhealthy, stereotype in the surgical domain where demonstrating resilience and emotional control is expected [9, 10]. Often, untoward incidents are attributed to technical lapses or inadequate preoperative or intraoperative decisions [10, 11], consequently emphasising technical aspects in discussions and sidelining the emotional aftermath [5, 10].

Recovering from SVS is subject to a multitude of influencing factors. Scott et al. [2] delineated six distinct stages of recovery: initial chaos and accident response, subsequent intrusive reflections, the restoration of personal integrity, enduring the inquisition, seeking emotional first aid, and ultimately progressing forward. Notably, the impact of SVS is influenced by personal, interpersonal, and environmental factors, which can either ameliorate or exacerbate its effects. For surgeons, the nature of complications, the presence of robust support systems, and the individual’s personality traits have been highlighted as key influencing factors [11–16]. As healthcare systems evolve and continue to be put under strain, the enhancement of service availability and accessibility aimed at mitigating intraoperative adverse events and the ramifications of intraoperative adverse events and SVS is of paramount importance, especially since many surgeons may not even be aware of their own distress and seek help [17].

Thus, this multicentre, cross-sectional study aimed to explore the degree to which the experience of an adverse event is common, as well as its impact, and factors affecting their responses and perceived support systems amongst surgeons locally. There are certain nuances within the Asian context which may make healthcare organizations more hierarchical [18] and influence the outcome and response to intraoperative adversities, encumbering individual surgeons across psychological, emotional, and physical domains. Through our findings, we hope to improve current understanding of the topic and inform further mitigation and resolution strategies when adverse events occur, ultimately aiding surgical professionals in managing them more effectively.

## Methods

This cross-sectional survey study is reported according to the STROBE (strengthening the reporting of observational studies in epidemiology) guidelines [19].

### Study participants

The survey link was distributed via official work email to all practising surgeons (including surgical residents-in-training) at four major teaching and academic hospitals in Singapore. The survey was conducted anonymously over a secured, electronic platform. The following specialties were represented in the survey: Breast Surgery, Cardio-Thoracic Surgery, Colorectal Surgery, General Surgery, Hand Surgery, Head & Neck Surgery, Hepatopancreaticobiliary Surgery, Maxillofacial Surgery, Neurosurgery, Obstetrics and Gynaecology, Orthopaedic Surgery, Ophthalmology, Ear, Nose and Throat (ENT)-Otorhinolaryngology, Paediatric Surgery, Plastic, Reconstructive and Aesthetic Surgery, Surgical Oncology, Trauma Surgery, Upper Gastrointestinal Surgery, Urology, Vascular Surgery. Responses were aggregated and de-identified to establish anonymity and encourage honest feedback. For the purposes of the study, we focused on intraoperative adverse events, defined as any inadvertent injury that occurs during the course of an operation. This working definition was communicated to all participants at the beginning of the survey.

### Questionnaire design

Through multiple team discussions, as well as consultation with a research psychologist and sociologist, iterations and reference to previous studies [11, 20–22], a 42-item web-based questionnaire was developed using a systematic closed and open approach focused on assessing five main domains: (1) Surgeon’s demographic, (2) Surgeon’s personal experience with adverse events and adverse events reporting, (3) Emotional and psychological impact of event, (4) Physical impact of event, and (5) Perceived support systems. The questionnaire was piloted with five doctors to refine its clarity and wording. The five doctors were not included in the final study sample. The full questionnaire may be found in Table S1. Depending on the nature of the closed-ended question, respondents could select a single answer, “check all that applies”, or rate using cursors on a 5-point Likert scale. Emotional and physical impact of the event was rated on a 5-point Likert scale, where 1 = not at all and 5 = very significant distress. Study responses and data were collected and managed using the FormSG electronic tool over two months, from June to August 2023. Three reminders were sent to eligible persons to complete the survey.

### Statistical analysis

Data were collated and analysed in Microsoft Excel and R 4.0.3 (R Foundation for Statistical Computing, Vienna, Austria). To find out differences in responses to intraoperative adverse events between genders and role (junior/senior residents as opposed to consultant/senior consultant/emeritus consultant), the data was stratified accordingly and analysed. Quantitative analyses of the closed-ended answers across surgeons for the different questions were performed, and the results reported included descriptive statistics such as median, range and percentages, where appropriate. Categorical variables were summarised using frequencies and percentages. Two-sample t tests and chi-square tests were used to compare continuous and categorical variables, respectively. Statistical significance was set at *p* < 0.05. In addition, logistic regression was performed to compare key outcomes against years of surgical experience. Lastly, the free-text statements were discussed iteratively and analysed qualitatively by the study team members, and verbatims were selected and reported to illustrate themes that emerged from the responses.

### Ethical approval

The study protocol was reviewed and approved by the Centralized Institutional Review Board (CIRB), reference number 2023/2279.

## Results

### Respondent demographics

Based on the nominal roll, a total of 341 surgeons received the survey, and 196 surgeons returned the survey, contributing to an overall response rate of approximately 57%. Out of the returned surveys, there were no incomplete or missing responses. Most respondents were male (*n* = 127, 55%), between 35 and 44 years old (*n* = 81, 41%) and holding Senior Consultant roles (*n* = 70, 36%). The mean number of years of surgical experience among the participants was 14.2 years (SD = 9.4). Majority of the respondents were from the following specialties: Orthopaedic Surgery (*n* = 35, 18%), Obstetrics & Gynaecology (*n* = 25, 13%) and Urology (*n* = 23, 12%). The aggregated demographics and characteristics of the study respondents are summarised in Table 1.

### Personal experience with intraoperative adverse events

The majority of respondents (*n* = 135, 69%) reported having dealt with adverse events in their career. In the past 12 months, 46 (34%) recalled encountering one adverse event, 2 (2%) recalled encountering two to five adverse events and 30 (22%) recalled encountering more than five adverse events (Table 2).

When asked about their history of reporting the incident to the hospital, 74 (38%) and 19 (10%) of responders who had encountered adverse events reported all or some of their adverse events to the hospital respectively (Table 2). Factors that made surgeons more likely to report the adverse event included poor patient outcomes (*n* = 94, 48%), high perceived severity (*n* = 87, 44%), high risk of litigation (*n* = 77, 39%) and prior case discussion at morbidity and mortality (M&M) rounds (*n* = 60, 31%). Other factors reported are detailed in Table 2.

Regarding the outcomes of these intraoperative adverse events, a substantial number resulted in harm to patients (*n* = 78, 40%), while others (*n* = 54, 28%) ended with no serious consequences. Other common outcomes included facing legal action (*n* = 15, 8%) and disciplinary actions or complaints (*n* = 11, 6%) against the surgeon.

### Physical impact

Based on self-reporting, the physical impact of adverse events had a mean impact score of 1.96 (SD = 1.15), as rated on a 5-point Likert scale (with a higher score implying greater impact). The most commonly reported somatic symptoms were headaches (*n* = 25, 13%), weight gain (*n* = 10, 5%), weight loss (*n* = 9. 5%), nausea (*n* = 9, 5%) and abdominal pain (*n* = 5, 3%), as seen in Table 3. Most respondents (*n* = 70, 52%) did not experience any physical impact. For respondents that experienced somatic symptoms, the physical sequelae were reported to last less than a week (*n* = 24, 18%), or between a week to a month (*n* = 24, 18%).

### Emotional and psychological impact

In contrast of the physical impact, the emotional impact of encountering adverse events was more pronounced, with participants rating a mean impact score of 4 ± 1.02 out of 5. The most commonly reported emotions faced were sadness (*n* = 123, 63%), guilt (*n* = 104, 53%), anxiety (*n* = 89, 45%), professional embarrassment (*n* = 41, 21%) and insomnia (*n* = 41, 21%), as seen in Table 3. For most respondents, the emotional sequelae lasted between a week to a month (*n* = 43, 32%) or one to six months (*n* = 36, 27%). Worryingly, 9 respondents (7%) reported that the emotional sequelae lasted for more than two years. Encountering adverse events also affected relationships between colleagues in 28 (20.7%) respondents.

Overall, 15 respondents (11%) reported negative effects from the event on subsequent clinical practice, such as defensive practice (*n* = 11, 74%), decreased job satisfaction (*n* = 7, 47%), and trauma (*n* = 7, 47%), as shown in Table 3. Conversely, a lasting positive effect was reported in a significant number of participants (*n* = 92, 68%). Positive effects included improved vigilance towards errors (*n* = 89, 45%), gaining new insight (*n* = 54, 28%), and becoming an advocate for patient safety (*n* = 22, 11%), as shown in Table 3.

### Perceived support systems

In the aftermath of an adverse event, 49 respondents (36%) expressed the need for a recuperative period away from work, however, only five respondents (10%) actually took time off, of which the majority took less than two weeks leave. A total of 25 respondents (18.5%) received emotional support subsequent to the incident, with the majority stemming from colleagues (*n* = 20, 10%), family members (*n* = 14, 7%), and their clinical supervisors (*n* = 13, 7%). Among these support mechanisms, colleagues, family, and clinical supervisors were perceived to be the most efficacious sources, as indicated by 39%, 20%, and 13% of the respondents respectively (detailed in Table 4).

Majority of the respondents did not receive counselling (*n* = 126, 93%). The main reason was that most participants did not deem counselling as necessary (*n* = 91, 72%). Other reasons include the perceived unavailability of counselling services (*n* = 37, 29%), concerns about the lack of confidentiality during the counselling process (*n* = 16, 13%), and the fear of negative judgement from colleagues (*n* = 14, 11%).

When asked to rank the utility of support systems, respondents reported that speaking to physicians who had experienced similar incidents (*n* = 90, 67%), family member (*n* = 68, 50%), and talking with the patient affected (*n* = 49, 36%) were the most salient support systems (Table 4). Other notable support mechanisms that respondents found helpful included self-reflection (*n* = 21, 11%), exercise (*n* = 17, 9%), reviewing of literature and video (*n* = 8, 4%) and seeking religious help (*n* = 7, 4%). In the future, respondents hoped that their support systems may involve supportive colleagues, peer support groups and coaching (*n* = 57, 30%), a more supportive work culture (*n* = 24, 13%), counselling (*n* = 11, 6%) and the allowance of time-off (*n* = 10, 5%) (Table 4). Particularly, a yearning for a more supportive institutional culture towards surgical errors was echoed by the following representative quotes:

*“More mentorship with senior doctors instead of a hierarchy. Every mistake seems like a huge sin… [it is] difficult to talk about things when the condemnation and judgement is very strongly felt.” (P1)*.

*“…for the higher-ups to take these incidents as learning experiences instead of looking for scapegoats or playing the blame game. Oftentimes, doctors are blamed and humiliated in front of the entire department at M&M meetings.” (P4)*.

*“Stronger doctor support [sic] without judgement…platform to seek guidance both legal and clinical without repercussions…stronger medical union and recourse amongst doctors. Also, if doctors are protected better, allowed to learn and move forward after each incident, they will be more forthcoming with their mistakes.” (P7)*.

**Table 1:** Summary of respondent demographics

Respondent demographic		n (%)
Gender		
	Male	127 (64.8)
	Female	69 (35.2)
Age		
	< 35	54 (27.6)
	35–44	81 (41.3)
	45–54	35 (17.9)
	55–64	19 (9.7)
	> 65	7 (3.6)
Relationship Status		
	Single	39 (19.9)
	Married	146 (74.5)
	Divorced	11 (5.6)
Role		
	Emeritus Consultant	5 (2.6)
	Senior Consultant	70 (35.7)
	Consultant	58 (29.6)
	Service Registrar^1^	13 (6.6)
	Resident^2^	50 (25.5)
Years of Experience (mean, SD)		14.18 ± 9.38
Specialty		
	Breast Surgery	9 (4.6)
	Cardio-Thoracic Surgery	1 (0.5)
	Colorectal Surgery	14 (7.1)
	ENT-Otorhinolaryngology	7 (3.6)
	General Surgery	10 (5.1)
	Hand Surgery	9 (4.6)
	Head & Neck Surgery	3 (1.5)
	HPB Surgery	5 (2.6)
	Maxillofacial Surgery	6 (3.1)
	Neurosurgery	3 (1.5)
	Obstetrics & Gynaecology	25 (12.8)
	Ophthalmology	4 (2.0)
	Orthopaedic Surgery	35 (17.9)
	Pediatric Surgery	6 (3.1)
	Plastic, Reconstructive & Aesthetic Surgery	11 (5.6)
	Surgical Oncology	6 (3.0)
	Trauma Surgery	2 (1.0)
	Undecided	10 (5.1)
	Upper Gastrointestinal Surgery	2 (1.0)
	Urology	23 (11.7)
	Vascular Surgery	5 (2.6)
Average Hours Worked per week (mean, SD)		59.65 ± 13.76
Average No. of Call Nights per week (mean, SD)		1.11 ± 0.82

^1^Service registrar include staff physician and service senior registrar who are not in a formal residency training programme

^2^Residents include junior and resident residents who are in a formal residency training

**Table 2:** Respondents’ experience with intraoperative adverse event(s)

Event Characteristic		n (%)
Experienced an Adverse Event		
	Yes	135 (68.9)
	No	61 (31.1)
Adverse Events in the past 12 months		
	0	57 (42.2)
	1	46 (34.1)
	2 to 5	2 (1.5)
	> 5	30 (22.2)
Incident Reported to the Hospital		
	No	42 (21.4)
	Some	19 (9.7)
	Yes	74 (37.8)
Factors Making Reporting More Likely		
	Poorer Patient Outcome	94 (48.0)
	Higher Perceived Severity	87 (44.4)
	Risk of Litigation	77 (39.3)
	Discussed at M&M rounds	60 (30.6)
	Patient and Family reactions	49 (25.0)
	Colleague Reactions	29 (14.8)
	Elective procedure	21 (10.7)
	Hierarchical Institution Culture	21 (10.7)
	None of the above	12 (6.1)
	Emergency Procedure	11 (5.6)
	All Complications need to be reported	5 (2.6)
	Preventable or Foreseeable Events	2 (1.0)
	Affecting Patient care or Safety	1 (0.5)
Serious Consequences		
	Harm to Patient	78 (39.8)
	No serious implications	54 (27.6)
	Legal action	15 (7.7)
	Disciplinary Action and Complaint	11 (5.6)
	Expectations of patient not met	1 (0.5)

Abbreviation: M&M = Morbidity and Mortality

**Table 3:** Physical, emotional and psychological sequelae from intraoperative adverse event(s)

Sequelae Characteristics		n (%)
Physical Impact (mean, SD)^1^		1.96 ± 1.15
Type of Physical Sequelae		
	None	80 (40.8)
	Headache	25 (12.8)
	Weight Gain	10 (5.1)
	Weight Loss	9 (4.6)
	Nausea	9 (4.6)
	Abdominal Pain	5 (2.6)
	Lethargy	3 (1.5)
	Low Appetite	2 (1.0)
	Tremor	2 (1.0)
	Psoriasis Flare	1 (0.5)
Duration of Physical Sequelae		
	1 week or less	24 (17.8)
	1 week − 1 month	24 (17.8)
	1–6 months	6 (4.4)
	6–12 months	6 (4.4)
	1–2 years	2 (1.5)
	> 2 years	3 (2.2)
Emotional Impact (mean, SD)^1^		4.00 ± 1.02
Type of Emotional Sequelae		
	Guilt	104 (53.1)
	Anxiety	89 (45.4)
	Sadness	72 (36.9)
	Embarrassment	41 (20.9)
	Insomnia	41 (20.9)
	Anger and Frustration	3 (1.5)
	Stress	2 (1.0)
	None	6 (3.1)
Duration of Emotional Sequalae		
	1 week or less	19 (14.1)
	1 week − 1 month	43 (31.9)
	1–6 months	36 (26.7)
	6–12 months	17 (12.6)
	1–2 years	9 (6.7)
	> 2 years	9 (6.7)
Relationships with Colleagues Affected		
	Yes	28 (20.7)
	No	99 (73.3)
Incident left a Positive Effect		
	Yes	92 (68.1)
	No	15 (11.1)
	I don’t know	25 (18.5)
Negative Effects		
	Defensive Practice	11 (73.3)
	Decreased Job Satisfaction	7 (46.7)
	Traumatizing	7 (46.7)
	Punitive Process	2 (13.3)
	Rethink Career Choice	2 (13.3)
	Affected Reputation	1 (6.7)
Positive Effects		
	Improved Vigilance to Errors	89 (45.4)
	Gain New Insight	54 (27.6)
	Advocate for Patient Safety	22 (11.2)
	Change Practice positively	3 (1.5)
	Patience and Anger management	1 (0.5)

^1^As reported using a 5-point Likert scale, with a higher score implying greater impact

**Table 4:** Perceived support systems

Support System		n (%)
Needed Time Off to Recover		
	Yes	49 (36.3)
	No	86 (63.7)
Actually Took Time Off to Recover		
	Yes	5 (10.2)
	No	44 (89.8)
How Much Time was Taken Off		
	< 2 weeks	4 (66.7)
	< 3 months	1 (16.7)
Emotional Support Received		
	Yes	25 (18.5)
	No	100 (74.1)
Who was Emotional Support Received from		
	Colleagues	20 (10.2)
	Family	14 (7.1)
	Clinical Supervisor	13 (6.6)
	Friends	11 (5.6)
	Faculty	4 (2.0)
	Religion	1 (0.5)
Emotional support was helpful		
	Yes	25 (96.2)
	No	1 (3.8)
Who was Most Helpful		
	Colleagues	52 (38.5)
	Family	27 (20.0)
	Clinical Supervisor	17 (12.6)
	Friends	10 (7.4)
	Faculty	8 (5.9)
	Hospital Administration	2 (1.5)
	Keep to Myself	2 (1.5)
	Pray to God	1 (0.7)
Received Counselling		
	Yes	1 (0.7)
	No	126 (93.3)
Reasons for No Counselling		
	Unnecessary	91 (72.2)
	Counselling Not available	37 (29.4)
	Afraid that information would not be confidential	16 (12.7)
	Afraid of negative judgement	14 (11.1)
	Afraid that malpractice insurance costs are affected	2 (1.6)
	No time	1 (0.8)
Most Important Support Mechanism		
	Speaking to physicians with similar incidents	90 (66.7)
	Family	26 (19.3)
	Speaking with patient affected	11 (8.1)
	Counselling	3 (2.2)
	Colleagues	3 (2.2)
	Religion	1 (0.7)
	Self	1 (0.7)
Second Most Important Support Mechanism		
	Family	68 (50.4)
	Speaking to physicians with similar incidents	36 (26.7)
	Speaking with patient affected	15 (11.1)
	Counselling	15 (11.1)
	Speaking with other patients	1 (0.7)
Third Most Important Support Mechanism		
	Speaking with patient affected	49 (36.3)
	Counselling	34 (25.2)
	Family	29 (21.5)
	Speaking to physicians with similar incidents	10 (7.4)
	Colleagues	6 (4.4)
	Speaking with other patients	3 (2.2)
	Vices	1 (0.7)
	Reading up on Similar Cases	1 (0.7)
	Time Off	1 (0.7)
Other Support Mechanisms		
	None	99 (52.7)
	Reflect and learn	21 (11.2)
	Exercise	17 (9.0)
	Discuss Case with Colleagues	10 (5.3)
	Literature and Video Review	8 (4.3)
	Time Off	8 (4.3)
	Religion	7 (3.7)
	Alcohol and Other Vices	6 (3.2)
	Repression	6 (3.2)
	Eat	6 (3.2)
	Hobbies	5 (2.7)
	Let Time Pass	5 (2.7)
	Family	3 (1.6)
	Meditation	3 (1.6)
	Discuss with Patient	2 (1.1)
Suggestions for Healthcare Organisations		
	None	88 (46.8)
	Supportive Colleagues, Peer Support Groups & Coaching	57 (30.3)
	Supportive Work Culture	24 (12.8)
	Counselling	11 (5.9)
	Time Off	10 (5.3)
	Legal Support	5 (2.7)
	Patient Advocate and Support Services	4 (2.1)
	Protocols	4 (2.1)
	Awareness of SVS	3 (1.6)

### Effects of role, years of surgical experience and surgeon gender on response to intraoperative adverse events

When stratified by gender, there was no significant difference in the incidence of adverse events in the past 12 months (*p* = 0.28), physical impact (*p* = 0.46), emotional impact (*p* = 0.37) or positive effect experienced after an adverse event (*p* = 1.00) (Table S2). When stratified by role (either junior/senior resident or consultant/senior consultant/emeritus consultant), there was no significant difference in the incidence of adverse events in the past 12 months (*p* = 1.00), physical impact (*p* = 0.08), emotional impact (*p* = 0.29), or positive effect experienced following an intraoperative adverse event (*p* = 0.15) (Table S3). Finally, a logistic regression analysis was conducted to examine the relationship between the occurrence of adverse events in the past 12 months and the years of surgical experience. The results revealed that the years of surgical experience did not explain the occurrence of adverse event in the past 12 months (odds ratio (OR) 0.986, 95% CI: 0.968–1.00). In another logistic regression that modelled the relationship between years of surgical experience and positive effect following an adverse event, a negative relationship between both variables was observed (OR 0.944, 95% CI: 0.944–0.998). The analyses can be found in Table S4.

## Discussion

This study sought to explore the degree to which adverse events are common, as well as the challenges and concerns that surgeons face within their professional environment, at four large tertiary hospitals in Singapore, a Southeast Asian country. Firstly, the occurrence of intraoperative adverse events was higher than initially expected, with the majority of respondents (*n* = 135, 68.9%) reported having dealt with adverse events in their careers and 46 (34.1%) in the past 12 months alone. Prior reports suggested an incidence of intraoperative adverse events and postoperative complications at 24% and 33% respectively [23]. Further research is required to more closely and prospectively examine the epidemiology of intraoperative adverse events, however, proper reporting of adverse events needs to be in place to facilitate such research. In our study, 21.4% of the respondents did not report the adverse event to the hospital while 9.7% only reported some of the incidents. Han et al. [11] shared similar sentiments, establishing notable reasons for poor reporting to be fear of litigation and reprisal and a lack of a clear working definition for reportable adverse events, and recommending adverse events be defined as any inadvertent intraoperative injury and severity, graded by scales such as those validated by Kaafarani et al. [24]. In addition, our study found that certain factors made a surgeon more inclined to report adverse events such as poorer patient outcomes, higher perceived severity and prior case discussion at M&M rounds. With these in mind, perhaps efforts to improve M&M rounds, as discussed later, could improve the reporting of adverse events.

These adverse events had a significant emotional and psychological toll on the affected surgeon, with some effects even lasting more than two years. Negative effects on subsequent clinical practice such as defensive practice, decreased job satisfaction was also reported by previous studies in this area [15, 16, 22, 25–27]. Commonly reported emotions in this study, including sadness, guilt, anxiety and embarrassment, was echoed by previous studies as well [10–12, 14, 16, 22, 25, 28]. Although not explored in this study, several factors may affect the extent of emotional sequelae. The heightened sense of personal responsibility in surgeons places them at risk of severe distress after their involvement in surgical complications, as self-criticism is a significant predictor of sadness in clinicians [29–32]. Other factors include elective procedures, severity of complications [12, 14, 16], the surgeon’s personality and experience [11–16]. While one might anticipate that a more senior surgeon would possess the ability to handle complications more effectively due to their enhanced maturity, confidence, and superior track record [11, 12, 15, 16], our findings have contradicted this view, with physical (*p* = 0.081) and emotional impact (*p* = 0.287) and the occurrence of intraoperative adverse events being comparable between junior and senior surgeons (*p* = 1.00). However, the role of supervision during adverse events, especially given that trainees typically operate under the guidance of more experienced surgeons, remains an area for further exploration. Our logistic regression demonstrated that an increase in years of surgical experience was associated with a less positive effect following an adverse event (OR: 0.944, 95% CI: 0.894–0.998, *p* = 0.0353), perhaps explained by suggestions that as surgeons advance in their careers, the weight of responsibility towards their patients also intensifies, causing surgeons to ruminate even over the most minor errors [12]. Moreover, as senior surgeons are more likely to perform more complex surgery, the impact and potential consequences of adverse events may not be comparable to simpler surgery performed by junior surgeons.

Another factor worth exploring is gender differences. Women, in particular, were found to be twice as likely to experience significant stress following adverse events [33], and they may be more susceptible to burnout, exacerbated by the additional demands of balancing work-home conflicts, as many women bear the dual responsibility of providing childcare within their households [33]. Similarly, our findings have contradicted this view, with the physical (*p* = 0.461) and emotional impact (*p* = 0.371) and the occurrence of intraoperative adverse events found to be similar between genders (*p* = 0.277). These factors necessitate further investigation, with the goal of tailoring interventions to assist surgeons in effectively coping with these challenges.

This study also sought to explore the coping mechanisms of surgeons and the barriers faced. Most commonly, surgeons preferred to discuss the adverse event with colleagues. This is consistent with previous studies that showed that most distressed healthcare providers seek support from peers rather than from family and friends [34–39]. Conversation with colleagues is empowering, as it alleviates intense emotions through reassurance and validation, especially since peers are more likely to share similar experiences [35, 38, 39]. This highlights the importance of fostering a culture of camaraderie and trust among colleagues since they are often the first line of support. Feedback from this study suggests that support from colleagues may be improved, with 57 (30.3%) respondents indicating interest in peer support groups and coaching. In existing literature, the sentiment is agreed upon [40, 41]. In fact, initiatives such as the RISE programme [42, 43], and a surgeon-led peer support programme developed by El Hechi et al. [44] have been proven effective. For coaching, training curriculums to deal with adverse events and how to deal with the reactions to complications were recommended by previous studies [16, 45]. Interestingly, since a majority of respondents indicated a positive takeaway with improved vigilance towards errors and advocating for patient safety, they may serve as coaches for other surgeons with adverse events. These programmes should be extrapolated and trialled in local contexts to improve outcomes.

Our findings also indicated an intriguing discrepancy: while only a single respondent reported receiving counselling after experiencing an intraoperative adverse event, a larger number identified counselling as a key support mechanism. This disparity prompts a deeper consideration of the barriers to accessing or accepting mental health support within the surgical profession. Despite many ranking counselling as one of the top support mechanisms and the grave emotional impact of intraoperative adverse events, the preponderance of study respondents, constituting 93.3% (*n* = 126), did not avail themselves of counselling services. The underlying factors behind this trend were multifaceted: foremost, a substantial number of participants, accounting for 72.2% (*n* = 91), held the perspective that counselling was not imperative in their situation. In addition, 29.4% (*n* = 37) highlighted the unavailability of counselling as a decisive impediment. Notably, concerns related to confidentiality during the counselling process deterred 12.7% (*n* = 16) of participants, while apprehensions regarding unfavourable evaluations from colleagues dissuaded 11.1% (*n* = 14) from seeking counselling support. In concordance with the literature, second victim programs are often underutilised due to fears over confidentiality [46] and perceived stigma associated with seeking help [47]. Surgeons, in particular, might perceive a stigma associated with acknowledging vulnerability, fearing professional repercussions or judgment from peers. Such cultural norms can significantly hinder the utilisation of counselling services, even when they are recognized as beneficial. This is made worse in surgery where the culture is often competitive and unsympathetic [9, 11, 14, 16]. Using M&M rounds as an example, it has been reflected that M&M rounds may be accusatory and hostile, ruining an opportunity for an open discussion and education [11, 14, 16, 17, 48]. It was noted that even when M&M meetings were used constructively, there was a disproportionate focus on the technical aspects of the adverse event and did not address the emotional needs of those affected by the event [10]. This reflects an unhealthy psychology and echoes a need to shift the current work culture from a hierarchical and punitive environment to one that encourages mentorship, open communication, learning from mistakes, and overall support for doctors’ well-being [49]. Moving forward, normalising emotional responses, encouraging disclosure, whilst making use of current platforms such as M&M meetings would be an optimal suggestion to cultivate a more positive work culture [10, 48]. In instances where surgeons had supportive environments, they shared that M&M meetings aided their learning [17], and they felt more comfortable speaking with their colleagues [11, 13, 15, 21, 22]. By enhancing the support network for surgeons and fostering a nurturing workplace atmosphere, we can enhance the notable positive outcomes following adverse events, or posttraumatic growth [50]. This may include heightened attentiveness to errors and the acquisition of fresh perspectives. Although the factors that facilitate posttraumatic growth when surgical mishaps happen remain dearth, it is hoped that such individuals may evolve into advocates for patient safety, while simultaneously championing the cause of providing psychological support for future surgeons who might encounter similar challenging situations.

### Limitations

This study is not without its limitations. First, it remains unclear whether a correlation exists between the severity of the adverse event and the likelihood of developing SVS. In this study, we defined an adverse event as any unintended injury occurring during surgery, yet this broad definition warrants further clarification. Specifically, it would be beneficial to distinguish between adverse events that were effectively managed and corrected, and those that escalated into more serious patient harm. The psychological and professional impact on the surgeon is likely to vary significantly between these scenarios. Second, this survey sampled only surgeons practising in Singapore, which presents challenges in generalising these findings to surgeons in other healthcare settings, systems, or different geographical areas where contextual and cultural variations may significantly influence the results. Third, the overall response rate was 57.5%, making non-response bias still a possibility. In the same vein, despite the anonymity of the survey, many of the respondents may not have disclosed their involvement in an adverse event due to perceived stigma or social desirability bias. As a result, the actual proportion of residents who experienced adverse medical events could be higher than what was reported in this study. Fourth, three specialties (Obstetrics & Gynaecology, Orthopaedic Surgery and Urology) accounted for significantly higher percentage returns than the other surgical specialties. This may reduce the representativeness of our findings. Unfortunately given the small sample size, we could not examine differences in results across the different specialties. For future studies it may be of interest to explore why and how surgeons from different specialties perceive adverse events. Fifth, we did not collect data on psychological morbidity (e.g. history of depression or anxiety) from our participants. Individuals with pre-existing mental health conditions might experience and react to stressors differently compared to those without such a history. The absence of this baseline information is a limitation of our study. Last but not least, the survey (including the emotional and physical impact scales) employed in this study has not been validated as there are currently no established and validated surveys specifically designed to assess the topic of surgeon well-being and support in the Asian context.

## Conclusion

In conclusion, this study highlighted the common occurrence of intraoperative adverse events, and its repercussions among surgeons in Singapore. It also looked at the support and coping mechanisms for surgeons following an intraoperative adverse event and sheds light on areas of improvement such as a shift in work culture, to leveraging existing platforms such as colleagues and M&M rounds. Many surgeons encounter intraoperative adverse events and they are compelled to learn from these at times distressing occurrences in the course of their career. This process could be eased by better support systems and capitalising on the accumulated experiences and sharing by senior colleagues who have had similar experiences. To move forward means to let go of the past and yet retain its positive aspects and lessons. This collective wisdom serves as a torch passed on to fellow professionals in the field, guiding them on their journey ahead.

### Electronic supplementary material

Below is the link to the electronic supplementary material.

Supplementary Material 1

## Data Availability

Due to CIRB stipulations, data are only available from the corresponding author on reasonable request, and for researchers who meet the criteria for access to confidential data. The criteria include Institutional Review Board (IRB) approval, data use approval and a Research Collaboration Agreement.
