# Supplementary material for: Intraoperative adverse events among surgeons in Singapore: a multicentre cross-sectional study on impact and support
Source: BMC Health Serv Res. 2024 Apr 24;24:512. doi: 10.1186/s12913-024-10998-x (PMC11040834; doi:10.1186/s12913-024-10998-x)
Supplement: Supplementary file 1 — Supplementary Material 1 [file 12913_2024_10998_MOESM1_ESM.docx]

**Supplementary Material**

Table S1: Survey questionnaire

| **Question** | **Options*** |
| --- | --- |
| Surgeon Demographic | |
| 1. Current place of work | Singapore General Hospital  KK Women’s and Children’s Hospital  Sengkang General Hospital  Changi General Hospital |
| 2. Gender | Male  Female |
| 3. Age | <35 years old  35-44 years old  45-54 years old  55-64 years old  >65 years old |
| 4. Relationship status | Single  Married  Partnered  Widowed  Divorced |
| 5. Current role | Junior resident  Senior resident  Service registrar/staff physician  Senior service registrar/senior staff physician  Associate consultant  Consultant  Senior consultant  Emeritus consultant |
| 6. Years of surgical experience in numbers (rounded up to the nearest whole number) |  |
| 7. Specialty | Breast surgery  Cardio-thoracic surgery  Colorectal surgery  General surgery  Hand surgery  Head and neck surgery  HPB surgery  Maxillofacial surgery  Neurosurgery  Obstetrics-gynaecology  Ophthalmology  Orthopaedic surgery  Otorhinolaryngology-head and neck  Paediatric surgery  Plastic, reconstructive and aesthetic surgery  SPRinT  Surgical oncology  Trauma  Upper gastrointestinal surgery  Urology  Vascular surgery  Undecided |
| 8. On average, hours worked (per week) in numbers |  |
| 9. On average, number of call nights (per week) in numbers |  |
| Surgeon’s Personal Experience with Adverse events and adverse events reporting | |
| 10. During your practice, have you ever made an error (technical or clinical decision making) that caused harm or had the potential to cause harm to a patient? | No  Yes |
| If Yes, | |
| 11. In the past 12 months, how many personal intraoperative adverse events do you recall? | 0  1  2-5  >5 |
| 12. Was the incident(s) reported to the hospital? | Yes  Some  No |
| 13. In your opinion, what factor(s) would make you more likely to report the incident?  (Check all that apply) | If it was an elective procedure  If it was an emergency surgery  A hierarchical institutional culture  Higher likelihood of litigation  Greater patient/family's reactions  Greater colleague's reactions  Higher perceived severity  Poorer patient outcome  Whether case discussed at morbidity and mortality rounds  None of the above  Others |
| 14. Relating to ANY of your past experience(s), to what extent did the incident result in serious consequences?  (Check all that apply) | Harm to patient  Legal action  Disciplinary action  Not applicable  Others |
| Emotional and Psychological Impact | |
| 15. How much of an emotional impact did the incident have on you?  From scale 1 (Not at all) to 5 (Very significant) | 5-Point Likert Scale |
| 16. What kind of emotional sequelae did you experience?  (Check all that apply) | Insomnia  Anxiety  Sadness  Guilt  Embarrassment  Not applicable  Others |
| 17. How long did these emotional sequelae last? | One week or less  One week to one month  One month to six months  Six months to twelve months  More than one year but less than two years  More than two years  Not applicable |
| Physical impact | |
| 18. How much physical suffering did the incident cause you?  From scale 1 (Not at all) to 5 (Very significant) | 5-Point Likert Scale |
| 19.What kind of physical suffering did you experience?  (Check all that apply) | Weight loss  Weight gain  Headache  Tremor  Nausea  Abdominal pain  Not applicable  Others |
| 20. How long did these physical sequelae last? | One week or less  One week to one month  One month to six months  Six months to twelve months  More than one year but less than two years  More than two years  Not applicable |
| 21. Did this incident affect your relationships with your colleagues/seniors? | Yes  No  Not applicable |
| Support systems | |
| 22. Did you feel you needed time off to recover from the incident? | Yes  No |
| 23. Did you actually take time off to recover from the incident? | Yes  No |
| 24. How much time did you take off? | Less than 2 weeks  Less than 3 months  Less than 6 months  Less than 1 year  More than 1 year  I have not returned to work |
| 25. Did you receive emotional support for the incident? | Yes  No  Not applicable |
| 26. Who did you receive emotional support from? | Check all that apply  Family  Friends  Clinical supervisor  Other colleagues/residents  Faculty  Hospital administration  Other patients  Not applicable  Others |
| 27. If yes, who did you receive emotional support from? | Family Friends Clinical supervisor Other colleagues/residents  Faculty  Hospital administration  Other patients  Not applicable  Others |
| 28. Was the emotional support helpful? | Yes No I don’t know Not applicable |
| 29. Who was the most helpful to talk to after the incident? | Family  Friends  Clinical supervisor  Other colleagues/residents  Faculty  Hospital administration  Other patients  Not applicable  Others |
| 30. Did you receive counselling? | Yes  No  Not applicable |
| 31. If no, why not?  (Check all that apply) | I didn't consider it necessary  I was afraid it would affect my malpractice insurance costs  I was afraid it would be judged negatively  I was afraid the information would not be kept confidential  I was afraid the information would remain in my permanent record  Counselling was not available to me  Others |
| 32. If yes, where did you receive counselling | At institution (by faculty) At institution (by external provider) Outside Institution Not applicable |
| 33. Was counselling helpful? | Yes No I don’t know Not applicable |
| 34. If no, why not? |  |
| 35. In your opinion, what is the MOST important support mechanism? | Family  Speaking with other physicians who have encountered similar incidents  Speaking with patient affected  Speaking with other patients  Counselling  Others |
| 36. In your opinion, what is the SECOND most important support mechanism? | Family  Speaking with other physicians who have encountered similar incidents  Speaking with patient affected  Speaking with other patients  Counselling  Others |
| 37. In your opinion, what is the THIRD most important support mechanism? | Family  Speaking with other physicians who have encountered similar incidents  Speaking with patient affected  Speaking with other patients  Counselling  Others |
| 38. What other action(s) do you take to alleviate the stress from the incident? | Open-ended |
| 39. What kind of support would you wish to see in your institution for such incidents? | Open-ended |
| 40. Did the incident have any positive effect on you as a physician? | Yes  No  I don't know  Not applicable |
| 41. If yes, in what way? | It helped me improve vigilance towards avoiding similar errors It helped me gain new insight It helped me become an advocate for patient safety NA Others |
| 42. If no, why not? | Too traumatizing Process was punitive Decreased Job Satisfaction Affected reputation It made me rethink my career choice My practice became more defensive Others |

Table S2: Key Outcomes Stratified by Surgeon Gender

|  | **Female (n=69)** | **Male (n=127)** | **p-value** |
| --- | --- | --- | --- |
| Intraoperative adverse events in the past 12 months (n, %) | 22 (50.0) | 56 (61.5) | 0.277 |
| Physical Impact (mean (SD)) | 2.07 ± 1.11 | 1.91 ± 1.17 | 0.461 |
| Emotional impact (mean (SD)) | 4.11 ± 0.99 | 3.95 ± 1.04 | 0.371 |
| Positive effect (n, %) | 30 (85.7) | 62 (86.1) | 1 |

Table S3: Key Outcomes Stratified by Surgeon Role

|  | **Registrar (n=50)** | | **Consultant (n=133)** | **p-value** |
| --- | --- | --- | --- | --- |
| Intraoperative adverse events in the past 12 months (n, %) | | 14 (58.3) | 63 (57.8) | 1 |
| Physical Impact (mean (SD)) | 3.71 (0.86) | | 4.10 (1.02) | 0.081 |
| Emotional impact (mean (SD)) | 1.75 (0.79) | | 2.03 (1.21) | 0.287 |
| Positive effect (n, %) | 17 (100.0) | | 74 (83.1) | 0.148 |
|  |  |  |  |  |

Table S4: Logistic Regression of Key Outcomes Stratified by Surgical Experience

|  | **OR** | **95% CI** | **p-value** |
| --- | --- | --- | --- |
| Intraoperative adverse events in the past 12 months | 0.968 | 0.933 - 1.00 | 0.0833 |
| Positive effect | 0.944 | 0.894 - 0.998 | 0.0353* |

* = Statistically significant, P<0.05
